# Selecting South American Popcorn Germplasm for *Bipolaris maydis* Resistance at Contrasting Nitrogen Levels

**DOI:** 10.3390/plants14030302

**Published:** 2025-01-21

**Authors:** Yure P. Souza, Gabriel M. B. Gonçalves, Julio C. G. Saluci, Rafael N. Almeida, Juliana S. Santos, Hércules S. Pereira, Rysley F. Souza, Ana Lucia R. Souza, Luana C. Vasconcelos, Marcelo S. Andrade, Antonio T. Amaral, Marcelo Vivas

**Affiliations:** 1 Laboratório de Engenharia Agrícola, Universidade Estadual do Norte Fluminense Darcy Ribeiro, Campos dos Goytacazes 28013-602, RJ, Brazil; yure_p-souza@hotmail.com (Y.P.S.); gabriel.goncalves@pq.uenf.br (G.M.B.G.); juliosaluci@gmail.com (J.C.G.S.); almeida.rna94@gmail.com (R.N.A.); herculeshsp@gmail.com (H.S.P.); rysleyfernandes@gmail.com (R.F.S.); rangelana.agro@gmail.com (A.L.R.S.); luanavasconcelos16@hotmail.com (L.C.V.); marcelojunior.a@hotmail.com (M.S.A.J.); 2 Laboratório de Melhoramento Genético Vegetal, Universidade Estadual do Norte Fluminense Darcy Ribeiro, Campos dos Goytacazes 28013-602, RJ, Brazil; julianasaltiresdossantos@yahoo.com.br (J.S.S.); amaraljr@uenf.br (A.T.A.J.)

**Keywords:** biotic stress, genetic resources, maize, plant breeding, plant diseases

## Abstract

Nitrogen (N) availability plays a crucial role in plant development. However, studies indicate that the pathosystem of pathogenic fungi, such as *Bipolaris maydis*, which causes Southern Corn Leaf Blight (SCLB) in popcorn, interacts with N availability. Therefore, this study seeks to select popcorn inbred lines (ILs), considering contrasting environments regarding N availability (low N—LN and optimal N—ON). For this, 90 ILs from 16 populations from tropical and temperate climates from South America were evaluated in five experiments using a randomized complete block design (three replications), with four common controls. From the tests, the level of severity of the ILs to SCLB was evaluated. Three trials showed greater severity in ON, one trial had higher severity in LN, and one trial did not show significant differences. However, the genotype x nitrogen level (GxN) interaction was always present. Of the 90 ILs, 73 showed resistance in both N levels, three only in LN, and four only in ON, while 10 were susceptible in both environments. On average, the lines were more susceptible in ON, and the observed GxN interactions indicate that there is a distinct behavior of the genotypes regarding the response to N in the soil, which reinforces the importance of selection in contrasting environments.

## 1. Introduction

Brazil, the second-largest global consumer of popcorn after the U.S., produced 260 thousand tons in 2018, with 85% consumed domestically, generating USD 628 million. By 2024, popcorn consumption is projected to reach 385 thousand tons, with a revenue of USD 850 million [1].

The effect of nitrogen (N) fertilization on the fungal pathosystem in host plants is an intensely studied subject in many crops, including maize. In most of the research, the interaction of disease severity with N levels stands out, causing a greater expansion of the disease in the leaf tissue at higher levels of the nutrient [2,3,4,5,6].

Nitrogen is one of the most important nutrients for plant development, having a direct effect on cell size and cell wall thickness [7]. Despite the fact that a higher grain yield under high N levels requires an increase in fungicide use to control diseases, the gain in productivity under N over-application exceeds the gain under low N availability, when evaluated in single hybrids [8]. However, this contributes to negative environmental impacts of agriculture regarding water and air pollution [9] as most of the N is not consumed by the plant [10]; these impacts are also due to the high use of fungicides, which are toxic to the ecosystem, producer, and consumer.

Popcorn has a disadvantage compared to common corn because it is more susceptible to foliar diseases, as shown in an evaluation of Southern Corn Leaf Blight (SCLB) foliar disease [11,12] caused by the etiological agent *Bipolaris maydis* (Y. Nisk. & C. Miyake) Shoemaker. Under conditions of high humidity and temperature, SCLB usually presents higher rates of severity; therefore, yield losses can be very high [13,14,15]. According to a survey carried out by Mueller et al. (2019) [16], SCLB is among the 10 most destructive diseases in the south of the USA, a region with a climate more conducive to the development of the disease, with estimated losses close to 18 tons in 2019 in the region.

Resistance to *B. maydis* involves qualitative and quantitative mechanisms. The pathogen exists mainly as two races, O and T, with race T historically linked to severe epidemics in maize hybrids carrying cytoplasmic male sterility. Among known resistance genes, *hm1* encodes an NADPH-dependent HC-toxin reductase, providing resistance against *Cochliobolus carbonum* [17], while the *rhm1* mutant confers SCLB resistance by reducing fungal sporulation [18].

The most efficient, safe, and economical way to control diseases, in general, is the continuous process of developing resistant genotypes [11], and in the case of the Southeast region of Brazil, which has a humid tropical climate with hot summers (Aw), SCLB is a concern for the development of popcorn cultivars due to its wide occurrence in developing genotypes [3,6,19,20,21].

Kurosawa et al. (2020) [3] performed the characterization of popcorn populations from different regions of South America adapted to both tropical and temperate climates, and identified those that showed greater resistance to SCLB under field conditions in northern Rio de Janeiro, Brazil. However, new inbred lines (ILs) were developed from new populations and included in this work.

Thus, this work aimed to analyze popcorn ILs originating from 16 South America populations and identify those with resistance to SCLB in contrasting environments regarding the nitrogen availability.

## 2. Results

**Figure 1:**
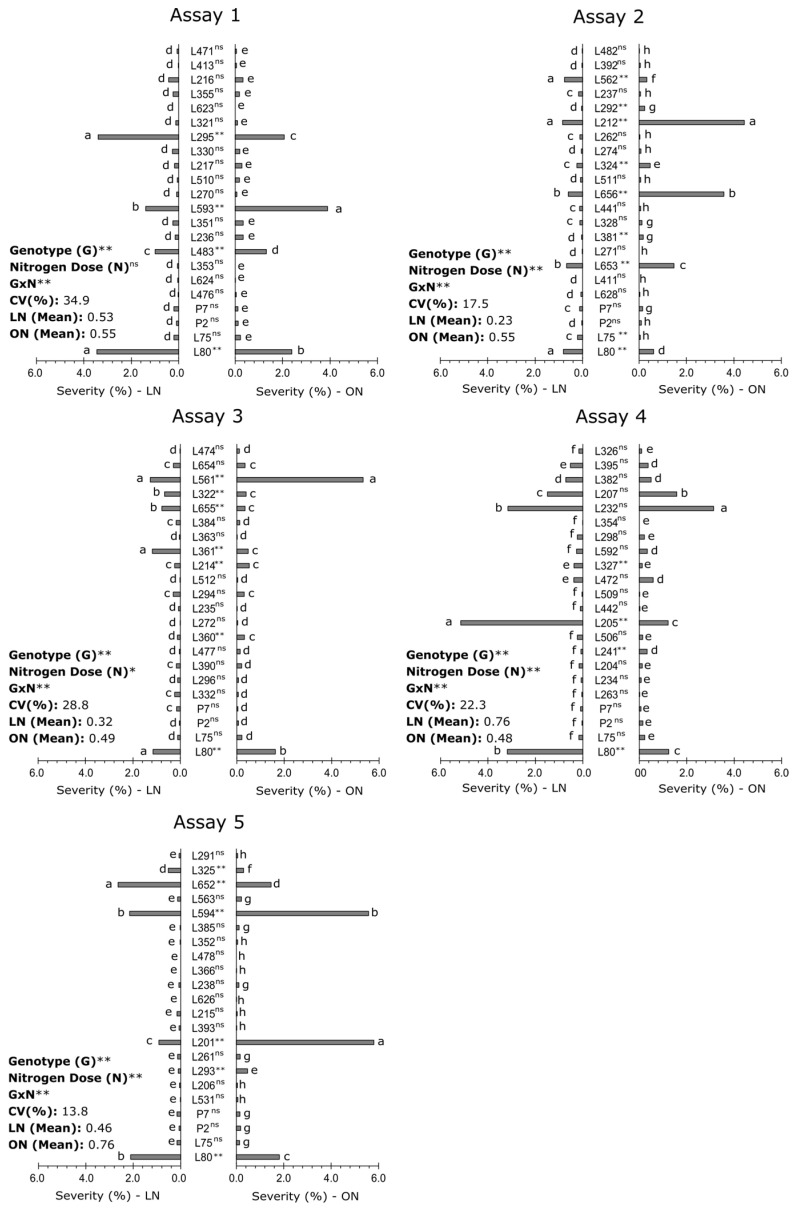
Severity means of Southern Corn Leaf Blight (*Bipolaris maydis*) in popcorn inbred lines (G) evaluated at low (LN) and optimal (ON) levels of nitrogen (N). The superscript characters in the sources of variation (G, N, and interaction G × N) refer to the statistical significance of the effects in the ANOVA: ns (not significant); * (significant at 0.05 error probability); and ** (significant at 0.01 error probability). Bars followed by the same letter in the same nitrogen condition and same assay did not differ statistically from each other according to the Scott-Knott algorithm at a 5% probability.

In all five trials with the popcorn inbred lines (ILs), there was a significant difference between the genotypes, with different groups being formed based on the severity means by the Scott-Knott algorithm. Except for Assay 1, all the other tests showed different means of severity between low N (LN) and optimal N (ON) environments, and only in Assay 4 did the environment with low nitrogen fertilization present a higher means of severity. Assay 1 was also the one with the highest coefficient of variation (34.9%), especially compared to Assay 2 and 5, which showed the lowest coefficient of variation (17.5% and 13.8%, respectively), emphasizing that all assays were equally dimensioned, having the same number of degrees of freedom and four common controls (Figure 1).

The genotype x environment interaction was present in all trials, evidencing the presence of genotypes that responded differently to the N increment. In Assay 1, the four ILs that presented the highest values of severity to SCLB in low and optimal nitrogen doses were the same (L295, L593, L483, and L80). However, L295 showed a high susceptibility, even at low N, with values very close to the control L80, which was considered susceptible [20]. The L593 IL showed a considerably greater increase in severity with N input. The other ILs showed low severity levels for SCLB.

In Assay 2, the mean severity between the ILs in the low-N environment was approximately 50% lower than in the high-N environment. In this environment, L562 and L212 stood out, which were in the same group as the susceptible control L80. The ILs L656 and L653 showed intermediate resistance in LN, while the other ILs showed similar averages to the controls, which were considered resistant: P7, P2, and L75. In ON, in the same assay, the lines L212 and L656 showed strong sensitivity to an increase in N, with much higher values of severity in relation to LN and in relation to the control L80.

Assay 3 also showed a high coefficient of variation of 28.8%. The mean severity in LN was 35% lower than in ON. However, the increase in severity under ON was mainly influenced by the means of L561 and L80, with L561 being the most susceptible in ON, with an average of 5.8% severity. In LN, lines L561, L361, and L80 were the most susceptible.

In Assay 4, L205, which showed high severity close to 5% in LN showed a severity of less than 1.5% in ON, a reduction of approximately 70%. Likewise, L80, which showed a 3% severity in LN showed a reduction of approximately 50% in severity in ON. L232 showed an average similar to L80 in LN and the highest severity in ON, but no significant difference was found between the means in both environments.

In Assay 5, the environment under a LN condition presented an approximately 40% lower mean severity compared to ON and a low CV (13.8%). In LN, the IL that presented the highest severity was L652, with the highest average, followed by L597 and L80. In the environment under ON, L201, which showed a moderate level of resistance in LN, showed a high level of susceptibility, superior to the other ILs. The severity of line L594 almost tripled in ON, and L652 showed a slightly lower severity than the susceptible L80.

Based on the performance of resistant and susceptible checks, three lines were considered resistant only under low nitrogen levels, four were considered resistant only under optimal nitrogen levels, seventy-three were resistant under both conditions, and ten were susceptible under both conditions (Table 1).

**Figure 2:**
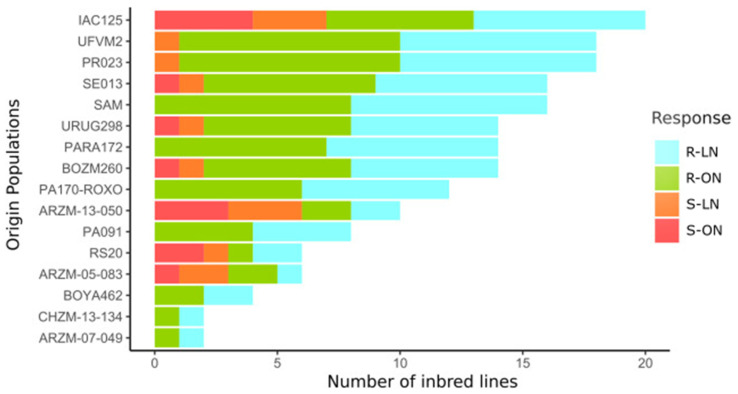
Number of popcorn inbred lines considered resistant (R) and susceptible (S) to *Bipolaris maydis* in each origin population, considering optimal nitrogen levels (ON) and low nitrogen levels (LN) in field experiments.

Figure 2 shows the response of each IL under the two N conditions. Each IL is represented by two results (IN and ON), meaning that ARM-07-049, represented by a single IL, reflects two outputs, for example.

Based on the percentage of ILs from each population that showed susceptibility to *B. maydis*, population ARZM 13 050, represented by five ILs, showed 60% occurrences of susceptibility in their ILs. Populations ARZM 05 083 and RS 20, both represented by three ILs, showed 50% susceptibility, while population IAC 125, represented by 10 ILs, showed 35% susceptibility. Populations that did not show any occurrence of susceptibility were PARA 172 (seven ILs), SAM (eight ILs), CHZM 13 134 (one IL), PA 170 ROXO (six ILs), BOYA 462 (two ILs), ARZM 07 049 (one IL), and PA 091 (four ILs) (Figure 2).

**Figure 3:**
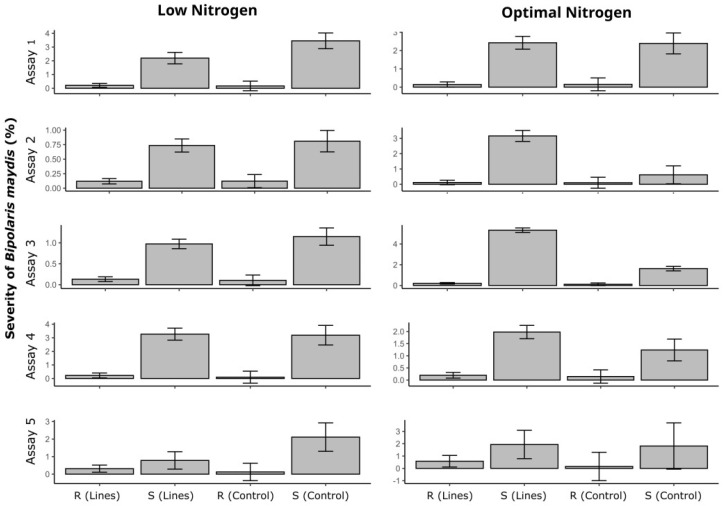
Averages of Southern Corn Leaf Blight severity (caused by *Bipolaris maydis*) for groups of popcorn inbred lines classified as resistant (R), susceptible (S), and controls (R and S) in environments under low and optimal nitrogen fertilization levels. The bars represent confidence intervals based on *t*-tests at a 5% probability of error.

**Figure 4:**
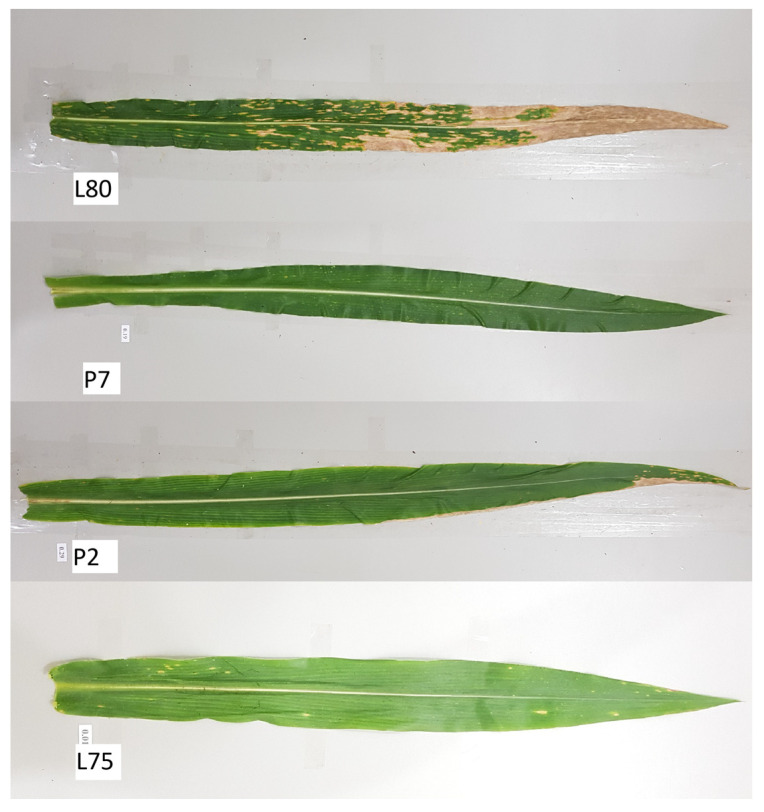
Detached leaves of the susceptible control (L80) and resistant controls (P7, P2, and L75) in response to *Bipolaris maydis*.

The classification of resistant and susceptible inbred lines in each experiment and N level reinforces the role of selections based on the controls, with overlapping confidence intervals between groups with the same resistance classification in most cases. Examples that did not follow this pattern were Assay 5 in the LN condition and Assay 2 and 5 in the ON condition, in which there were overlapping confidence intervals between treatments and resistant checks and the susceptible IL L80 (Figure 3). In cases such as Assays 2, 3, and 4, in the ON condition, the groups of genotypes classified as susceptible presented significantly higher severity than the susceptible control L80, indicating high susceptibility in the optimal environment of N fertilization for these genotypes (Figure 3). Figure 4 shows the infection pattern of the control treatments, namely, the susceptible (L80) and resistant (P2, P7, and L75) controls, which served as reference for classifying the lines across experiments.

## 3. Discussion

The present work, which evaluated popcorn inbred lines (ILs) for resistance to Southern Corn Leaf Blight (SCLB) based on the severity of foliar symptoms and contrasting levels of nitrogen (N) fertilization, was able to identify lineages and parents that are candidates for sources of alleles that are resistant to SCLB. Most of the genotypes originated from populations developed in eight different countries, characterized by yellow grain coloration and pearl-shaped grains. These populations, which gave rise to the genotypes, are partly maintained by Brazilian research institutions and largely by the International Center for Maize and Wheat Improvement (CIMMYT) (Table 1), a key global source of tropical and temperate maize germplasm [22,23].

Considering the complexity of evaluating diseases in the field and based on diagrammatic scales, the five assays carried out with different sets of ILs showed medium to high levels of reliability, in comparison to recent works. Bhandari et al. (2017) [11] obtained a coefficient of variation (CV) of 59% in the area under the curve of SCLB disease progress, while Saluci et al. (2020) [6] obtained CVs of 54% and 28% in the first and second evaluations, respectively. Furthermore, in general, the effects of genotype, N level, and the GxN interaction were highly significant (Figure 2). However, as these are tests that relied on the natural occurrence of the pathogen, it is possible that the results will not be reproduced in the same way, depending on the environmental conditions and the presence of the pathogen, as can be seen in other studies that considered more than one season [20,24].

Even under conditions of natural inoculation, the inference about the potential of genetic response to the occurrence of *B. maydis* was possible given that the averages of disease severity were close between assays, mainly in ON. The possibility of working with reliable disease assessment, even under natural pathogen inoculation, occurs in areas where there is a history of disease occurrence and constant cultivation [25] as well as the adaptation of the pathogen to survive in the area [26], as is the case in this study [2,20].

The higher level of nitrogen fertilization contributed to the increase in SCLB severity in four of the five experiments. This difference corresponds to what was reported in a review article by Snoeijers et al. (2000) [27], in which the authors concluded that the additional supply of nitrogen facilitates the acquisition of N by the pathogen, causing more severity in these plants in relation to plants under nitrogen limitation. Huber et al. (1974) [7] argued that the form of nitrogen available can influence the severity of the disease (nitrate or ammonia), and this will depend on the infection strategy of the pathogen. In the present study, however, nitrogen was provided in the form of urea CO(NH2)2, making both the absorbable forms of nitrate and ammonia available to the plant, which may have influenced the increase in severity in some genotypes.

The presence of interaction between the evaluated genotypes and the level of N shows the difference in the response of the plant in relation to fertilization and, consequently, in relation to the interaction of the pathogen with the host under conditions of low and high N, as already reported by Kurosawa et al. (2021) [2]. At contrasting levels of fertilization, genotypes can be efficient or non-efficient in the use of the input under low availability or be responsive or non-responsive under high availability, as observed in several works [28,29,30,31].

In a study carried out by Kurosawa et al. (2020) [3], populations ARZM 07 049 and PARA 172 were considered resistant, and this work confirmed the presence of resistance alleles in both populations based on the ILs extracted from them. In a similar study carried out by Kurosawa et al. (2021) [2], populations ARZM 05 083, BOZM 260, URUG 298, and IAC 125 were considered resistant to SCLB; however, the resistance that was broadly observed in the parent populations was not observed in the ILs (Figure 2).

Saluci et al. (2020) [6] performed a greenhouse experiment and evaluated 78 ILs in S4 from the same populations used in the present study. The ILs, inoculated at the V4 stage, with the best performances were the ones originating from populations SE 013, SAM, PR 023, PARA 172, PA 170 ROXO, PA 091, CHZM 13 134, BOZM 260, and ARZM 13 050. In the results obtained in this study and presented in Figure 3, it can be observed that the mentioned populations were considered good parents, with low occurrences of susceptibility to SCLB, except for population BOYA 462, which was not considered as a source of resistance to SCLB by the author.

Field confirmation of the results obtained in the greenhouse is a fundamental step in classifying the genotype in terms of its resistance, which may or may not be correlated. Furthermore, the preliminary study conducted by Saluci et al. (2020) [6] in S4 highlighted another important point in relation to the development of ILs, in which S4 and S7 showed a high correlation, as underlined by Hallauer et al. (2010) [32].

The selection of populations to obtain ILs begins with the characterization of the possible parents, which should concentrate on favorable alleles for the characteristics of interest [32]. However, obtaining lines with the best combinations is not a simple task, and as observed in this work, the correlation between parents and progenies will not necessarily be perfect, especially when considering inbred popcorn progenies, in which the depression is more accentuated due to its allogamous nature and contrasting environmental conditions regarding the availability of N.

In this more advanced stage of IL evaluation, it was possible to identify 73 lines that showed high or moderate resistance, which could be included in subsequent stages of the breeding program. According to Feher (1991) [33], preliminary evaluations of lines are essential for carrying out studies on combining ability using a reduced number of hybrids combinations. Furthermore, a study related to the genetic control of resistance to SCLB in PGB/UENF, conducted by Santos et al. (2019) [21], indicated that the non-additive effects are superior to the additive effects on the incidence and severity of SCLB, indicating that the production of hybrids may result in even more expressive gains in resistance in the following stages. The different origins of the ILs (16 unrelated populations) are another factor that may favor the exploration of non-additive effects on resistance to SCLB within the breeding program.

## 4. Materials and Methods

Ninety inbred lines (ILs) from the Popcorn Germplasm Bank of the Universidade Estadual do Norte Fluminense (PGB/UENF), Brazil, were evaluated. The ILs were developed from 16 populations that originated in the Americas, both in tropical and subtropical climates, all of which have yellow pearl-shaped grains (Table 2).

The 90 ILs were randomly divided into five distinct groups, containing 18 ILs each, and evaluated separately in a randomized complete block design with three replications. Each of the five experiments contained four controls—P7, P2, L75, and L80—these being ILs well characterized in previous works for SCLB severity, yield, popping expansion, and other diseases [3,6,20,29], with only L80 considered susceptible.

The experimental plots consisted of a row of 3.00 m with a spacing of 0.90 m between rows and 0.20 m between plants. Three seeds were sown per hole, and 21 days after emergence, thinning was performed, leaving only one plant per hole, resulting in a stand of 55,555 plants per hectare.

**Figure 5:**
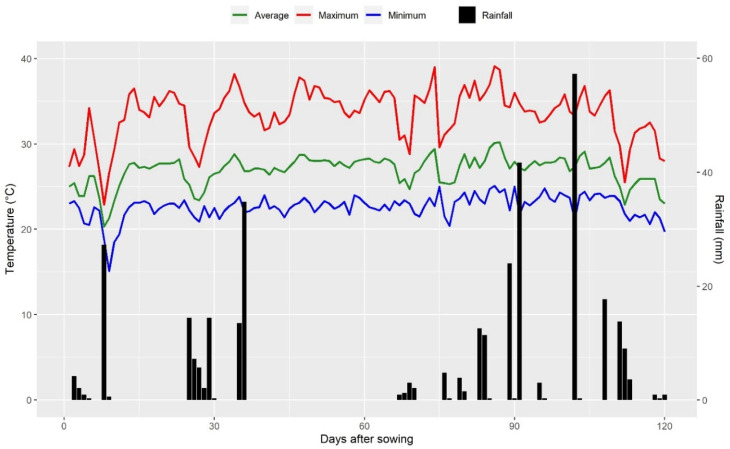
Precipitation (mm) and maximum, average, and minimum temperatures (°C) observed during the experimental period in the 2018–2019 crop year. Source: National Institute of Meteorology/Brazil (INMET).

The experiments were conducted during the 2018/19 crop year in two contrasting environments regarding nitrogen availability (optimal and low N) at the Experimental Station of Colégio Estadual Agrícola Antônio Sarlo, in Campos dos Goytacazes, RJ (latitude: 21°42′48″ S, longitude: 41°20′38″ W, and altitude: 14 m). The climate of this municipality is characterized as humid tropical (Aw), with hot summers and mild winters, according to the Köppen classification [34]. During the experimental period, an average temperature of 26.9 °C and average rainfall of 291 mm were observed (Figure 5). Before implementing the experiments, chemical analyses of the soil were carried out to characterize the cultivated soil (Table 3), and its results were interpreted. The estimated pH is ideal for maize cultivation (pH 6.0), the phosphorus (P) content was high, and the potassium (K) content ranged from high to very high. The soil organic matter content was estimated by spectrophotometry and was classified as low.

### 4.1. Characterization of Environments in Relation to Nitrogen Availability

The strategy adopted to differentiate between the environments regarding the level of nitrogen availability was adopted from previous studies [29,35,36], which followed the approach described by Gallais and Hirel (2004) [37], who reported a 40% yield reduction under low nitrogen availability. This differentiation was also aligned with the soil analysis. The basal dressing was applied in both environments, with 32 kg ha^−1^ of nitrogen added. In the environment with optimal N availability (ON), topdressing fertilization was carried out twice at the stages of four (V4) and six (V6) fully developed leaves, with the application of 118 kg ha^−1^ of N. In the environment with low availability of N (LN), the topdressing fertilization consisted of 28.5 kg ha^−1^, equivalent to 25% of that carried out in the environment with the optimal dosage of N. For both environments, urea was used as a source of nitrogen.

### 4.2. Evaluated Traits

For the evaluation of *B. maydis*, four alternate plants were identified within each experimental unit, disregarding the four plants at the beginning and the four plants at the end. The assessment considered the natural occurrence of the disease in an experimental area exclusively dedicated to maize cultivation, with a known history of *B. maydis* outbreaks. The diagrammatic scale proposed by James (1971) [38] was used. The scale is a widely used tool in plant pathology for estimating disease severity as a percentage. It relies on visual references representing different levels of severity, helping to standardize assessments and reduce subjectivity in quantifying the affected plant area. The evaluation of the severity of *B. maydis* was carried out 10 days after female flowering in 50% of the plots; this was performed on the leaf attached to the first ear on two occasions, with an interval of two weeks, and an average was taken.

### 4.3. Statistical Analysis

Initially, individual analysis of variance was performed for environments with optimal and low nitrogen levels within each trial. Subsequently, a joint analysis of variance was performed in order to determine possible interactions between genotypes and nitrogen levels.

The joint analysis of variance was performed according to the following statistical model:

Y_ijk_ = μ + B⁄A_jk_ + A_j_ + G_i_ + GA_ij_ + ε_ijk_,

where the observation Y_ijk_ is the i-th genotype in the j-th environment in the k-th block; μ is the general constant; B⁄A_jk_ is the effect of the k-th block within the j-th environment (level of N); A_j_ is the fixed effect of the j-th environment (level of N); G_i_ is the fixed effect of the i-th genotype; GA_ij_ is the fixed effect of the interaction between the i-th genotype and the j-th environment; and ε_ijk_ is the effect of experimental random error associated with the observation Y_ijk_, with NID (0, σ^2^).

To evaluate the general level of accuracy of the classification of the ILs, an analysis of variance (ANOVA) was performed for each assay and N level. The means were grouped using the Scott-Knott algorithm at a 5% probability. Confidence intervals for the resistant and susceptible IL groups were obtained based on *t*-tests, also at a 5% probability of error.

All statistical analyses were performed with the aid of the computational programs R version 4.3.1 (https://cran.r-project.org/bin/windows/base/ (accessed on 10 January 2025)) [39] and GENES (https://arquivo.ufv.br/genetica/WebSite1/Default.aspx (accessed on 10 January 2025)) [40].

## 5. Conclusions

The selection of inbred lines based on isolated experiments containing common controls was not restrictive, with a high number of genotypes being considered resistant. However, the greater number of selected lines allows other important characteristics, such as yield, popping expansion, and other diseases, to be considered more easily for the selection of parents for diallel crosses.

On average, the lines were more susceptible in an environment with high nitrogen availability. However, the observed genotype x nitrogen level interactions indicate that there is a distinct behavior of the genotypes in terms of interaction with the pathogen in relation to nitrogen uptake, reinforcing the importance of selection in contrasting environments.

## Figures and Tables

**Table 1:** Classification of popcorn inbred lines for resistance to *Bipolaris maydis* in environments under low (LN) and optimal (ON) nitrogen fertilization.

Resistant Only in LN					Resistant Only in ON			
L201	L593	L653			L655	L562	L322	L361
**Resistant in LN and ON**					**Susceptible in LN and ON**			
L204	L270	L328	L390	L506	L205	L207	L212	L295
L206	L271	L330	L392	L509	L594	L652	L483	L656
L214	L272	L332	L393	L510	L561	L232		
L215	L274	L351	L395	L511				
L216	L291	L352	L411	L512				
L217	L292	L353	L413	L531				
L234	L293	L354	L441	L563				
L235	L294	L355	L442	L592				
L236	L296	L360	L471	L623				
L237	L298	L363	L472	L624				
L238	L321	L366	L474	L626				
L241	L324	L381	L476	L628				
L261	L325	L382	L477	L654				
L262	L326	L384	L478					
L263	L327	L385	L482					

**Table 2:** Popcorn inbred lines (S7) from tropical (*) and temperate (**) regions and their respective source information.

Inbred Lines	N	Origin Populations	Country	Institution	References (Accessed on 10 November 2024)
L201 to L217	10	IAC 125	BR *	IAC	https://doi.org/10.4025/actasciagron.v46i1.62929
L232 to L241	7	BOZM 260	BOL**	CIMMYT	https://doi.org/10.18730/GPAF*
L261 to L274	7	PARA 172	PRY **	CIMMYT	https://doi.org/10.18730/GCFPQ
L291 to L298	7	URUG 298	URY **	CIMMYT	https://doi.org/10.18730/GJQSS
L321 to L332	9	UFVM 2—Barão de Viçosa	BR *	UFV	http://arquivo.ufv.br/dft/milho/UFVM2.htm
L351 to L366	9	PR 023	BR *	UENF	-
L381 to L395	8	SAM	USA **	UENF	-
L411	1	CHZM 13 134	CHL **	CIMMYT	https://doi.org/10.18730/GTMQG
L413 and L506 to L512	6	PA 170 ROXO	PRY **	UENF	-
L441 and L442	2	BOYA 462	COL *	CIMMYT	https://doi.org/10.18730/GBF2F
L471 to L483	8	SE 013	BR *	UENF	-
L531	1	ARZM 07 049	ARG **	CIMMYT	https://doi.org/10.18730/GQJGJ
L561 to L563	3	ARZM 05 083	ARG **	CIMMYT	https://doi.org/10.18730/H19ST
L592 to L594	3	RS 20	BR *	IPAGRO	
L623 to L628	4	PA 091	BR *	UENF	-
L652 to L656	5	ARZM 13 050	ARG **	CIMMYT	https://doi.org/10.18730/H1C4V

IAC—Instituto Agronômico de Campinas (SP-Brazil); UFV—Universidade Federal de Viçosa (MG-Brazil); UENF—Universidade Estadual do Norte Fluminense (RJ-Brazil); IPAGRO—Instituto de Pesquisa Agronômica (RS-Brazil); and CIMMYT—Centro Internacional de Mejoramiento de Maíz y Trigo (TXM—México).

**Table 3:** Soil chemical attributes for 0–10 and 10–20 cm deep layers in the experimental area of Colégio Estadual Agrícola Antonio Sarlo, in Campos dos Goytacazes, RJ.

Soil Layers	pH	P	K	Ca	Mg	Al	H + Al	Na	C	MO
	H_2_O	mg/dm^−3^	mmol_c_/dm^−3^						g dm^−3^	
0–10 cm	5.9	27	3.3	15.8	8	0	28.5	1.1	12.4	21.3
10–20 cm	5.8	28	2.4	17.6	8.6	0	38.1	0.8	13.4	23.1

MO—Soil organic matter content.

## Data Availability

Data is contained within the article.
